# Genome-dependent chromosome dynamics in three successive generations of the allotetraploid *Festuca pratensis* × *Lolium perenne* hybrid

**DOI:** 10.1007/s00709-014-0734-9

**Published:** 2014-12-06

**Authors:** Tomasz Książczyk, Elżbieta Zwierzykowska, Katarzyna Molik, Magdalena Taciak, Paweł Krajewski, Zbigniew Zwierzykowski

**Affiliations:** 1 Department of Environmental Stress Biology, Institute of Plant Genetics of the Polish Academy of Sciences, Strzeszyńska 34, 60-479 Poznań, Poland; 2 Department of Biometry and Bioinformatics, Institute of Plant Genetics of the Polish Academy of Sciences, Strzeszyńska 34, 60-479 Poznań, Poland; 3 Present Address: Department of Plant Genetics, Breeding and Biotechnology, West-Pomeranian University of Technology, Słowackiego 17, 71-434 Szczecin, Poland; 4 Present Address: Poznań Plant Breeders Ltd., Wiatrowo Plant Breeding Branch, Wiatrowo 16, 62-100 Wągrowiec, Poland

**Keywords:** Chromosomal rearrangements, *Festuca* × *Lolium* hybrid, GISH, rDNA-FISH, 5S rDNA, 35S rDNA

## Abstract

We focus on the identification of complete and recombined ribosomal DNA-bearing chromosomes, and the dynamics of chromosomal number and position of ribosomal DNA (rDNA) loci in the F_2_-F_4_ generations derived from the F_1_ hybrid of *Festuca pratensis* Huds. (2*n* = 4*x* = 28) × *Lolium perenne* L. (2*n* = 4*x* = 28). *Lolium* genomic DNA and rRNA genes were mapped by means of genomic and fluorescence in situ hybridization (GISH and FISH). The results revealed that plants of the three generations share various rDNA loci profiles with chromosome structural changes, possibly as a result of chromosomal inter- and intra-rearrangements. We observed an asymmetrical variation in the number of recombinant arms with and without rDNA loci between parental genomes. The *Lolium* genome was more affected by rearrangements in arms with rDNA loci, while *Festuca* was more affected in arms without them. Statistically significant differences between *L. perenne* and *F. pratensis* genomes concerned the number of recombined chromosomes without rDNA, and the number of recombined rDNA-bearing chromosomal arms of marked chromosomes, showing a tendency of *F. pratensis* genome-like chromosomes to be less stable, compared with *L. perenne*. We postulate a novel genome-dependent range and type of chromosome variation in plants of the F_2_-F_4_ generations derived from *F. pratensis* × *L. perenne* hybrid.

**Electronic supplementary material:**

The online version of this article (doi:10.1007/s00709-014-0734-9) contains supplementary material, which is available to authorized users.

In the study of different plant genomes, including cultivars and inter-generic hybrids of the *Festuca-Lolium* complex, the possibility to identify mitotic and meiotic chromosomes is of major importance. Modern cytogenetic analyses, such as fluorescence and genomic in situ hybridization (FISH and GISH) techniques, have been widely used to resolve many processes of chromosome evolution, including structural rearrangements (Levin 2004), as well as extensive studies on phylogenetic and genomic relationships (Robledo et al. 2009), and to enhance our knowledge of plant genome structure and differentiation (D’Hont 2005; Maluszynska and Hasterok 2005; Cai et al. 2006; Zwierzykowski et al. 2008; Wolny et al. 2011; Wan et al. 2012; Chacón et al. 2012). Using a combination of double FISH with 5S and 35S ribosomal DNA (rDNA) probes, chromosome morphology and rDNA loci patterns have been described in *Festuca pratensis* (Thomas et al. 1997; Harper et al. 2004; Książczyk et al. 2010) and *Lolium perenne* (Thomas et al. 1996; Książczyk et al. 2010; Rocha et al. 2014), as well as in various amphiploid and introgression forms of *Festulolium* (Kopecký et al. 2006; Kosmala et al. 2006; Książczyk et al. 2010, 2012; Harper et al. 2011).

Contrasting patterns of genome organization in *Festuca* and *Lolium* species, involving chromosome substitution and homoeologous chromosome pairing, numerical and structural chromosome instability have already been observed in several generations of inter-generic *Festuca* × *Lolium* hybrids (Zwierzykowski et al. 1998, 2006, 2012). Studies of genome instability in the *Festuca*-*Lolium* complex has differentiated genetically close genomes such as *Lolium multiflorum* and *F. pratensis* (Thomas et al. 1994; Zwierzykowski et al. 1998; Kopecký et al. 2006; Kosmala et al. 2006), *L. perenne* and *F. pratensis* (King et al. 1998; Zwierzykowski et al. 2006, 2012), and *L. multiflorum* and *Festuca arundinacea* (Humphreys and Pašakinskienė 1996). The application of GISH/FISH in F_1_ hybrids of *F. pratensis* × *L. perenne* allowed the identification of *L. perenne* chromosome 3 and *F. pratensis* chromosomes 2 and 3 (*Lolium* and *Festuca* chromosomes were numbered according to Thomas 1981). This approach revealed variation in the number and position of rDNA sites, which can be easily monitored in these hybrids (Książczyk et al. 2010).

Numerous arrangements of rDNA chromosomal patterns within species were revealed in cultivars of *Festuca* spp. and *Lolium* spp. (Thomas et al. 1996, 1997, 2001; Harper et al. 2004). Moreover, it was shown by Książczyk et al. (2010) that variation in the number of 5S rDNA sites (gain/loss) occurred even among individuals derived from the same cultivar of *F. pratensis*. Pedrosa-Harand et al. (2006) also showed variation in the number of 35S rDNA loci among individuals of the same *Phaseolus vulgaris* accession. In F_1_ hybrids of *F. pratensis* × *L. perenne*, a new distally and interstitially located locus of 5S rDNA was observed (Książczyk et al. 2010; T. Książczyk and K. Molik, unpublished data), while in the tetraploid BC_1_ plants obtained from crosses of F_1_ hybrid *F. pratensis* × *L. perenne* into *L. perenne*, only a distally located new locus of 5S rDNA was found (Książczyk et al. 2012). The appearance of a distally located 5S rDNA site in hexaploid *Festuca gigantea* (Thomas et al. 1997), diploid *Festuca drymeja* (Harper et al. 2004), and diploid and tetraploid *F. pratensis* (Książczyk et al. 2010) may suggest its common distribution within *Festuca* species; however, the origin and extent of such a variation still remains unclear.

The present study aimed at characterizing the chromosomal number and position of rDNA sites, as well as mitotic chromosome behavior, in three successive generations, F_2_-F_4_, derived from F_1_ hybrid of *F. pratensis* (4*x*) × *L. perenne* (4*x*). As reported before, a cytogenetic examination of synthetic allotetraploid F_1_ hybrid of *F. pratensis* × *L. perenne* revealed various numbers of 5S and 35S rDNA sites (Książczyk et al. 2010). It was later showed by Książczyk et al. (2012) that the *L. perenne* chromosome 3 (5S + 35S rDNA) and *F. pratensis* chromosome 2 (35S rDNA) and 3 (5S rDNA) are involved in recombination, showing rearrangements in the BC_1_ plants. To the best of our knowledge, however, little is known about parental chromosome identification in the *Festuca-Lolium* complex, or any precise monitoring of recognized and unrecognized rearranged chromosomes of both parental genomes. We deal with this novel aspect in the present paper.

## Materials and methods

### Plant material

Tetraploid hybrids of *F. pratensis* (Fp) × *L. perenne* (Lp) (2*n* = 4*x* = 28, described here as Fp × Lp) were generated by inter-crossing autotetraploid forms of both species. *F. pratensis* Huds. (2*n* = 4*x* = 28) spontaneous tetraploid plants, obtained from twin seedlings of diploid cultivars, were used as the female parent, and *L. perenne* L. (2*n* = 4*x* = 28) as the male parent (Zwierzykowski et al. 2006). Four partially fertile female and male F_1_ hybrids were inter-crossed under controlled conditions, and the F_2_ progeny was generated. Generations F_3_-F_4_ were obtained by inter-crossing of 150 genotypes in control conditions. In this work, 30 randomly chosen plants (ten per each generation) with the tetraploid (2*n* = 4*x* = 28) number of chromosomes were used for cytogenetic analyses. In general, tetraploids comprised 66.7 % of plants studied (Z. Zwierzykowski, unpublished data). The cultivars of *F. pratensis* (4*x*), *L. perenne* (4*x*), and F_1_ hybrids of *F. pratensis* (4*x*) × *L. perenne* (4*x*) were previously studied in order to determine the number and position of rDNA sites (Książczyk et al. 2010; Online Resource S1). Due to the inter-cross of four F_1_ hybrids to produce the F_2_ progeny, a hypothetical model of F_1_ karyotypes was presented, considering a theoretical rDNA loci pattern (Online Resource S1). All four F_1_ plants used for hybridization in situ experiments had various rDNA loci patterns; hence, based on the number and position of their rDNA loci (four to five sites of 5S rDNA and seven to nine sites of 35S rDNA), we expected to observe seven to nine sites of 35S rDNA, consisting of both homologues of Fp chromosome 2 with large 35S rDNA sites, and 7 undifferentiated Lp chromosomes (besides both homologues of chromosome 3) with 35S rDNA, 2 large interstitial 5S rDNA sites in both Fp homologues of chromosome 3, 2 large interstitial 5S rDNA sites in the short arms of both Lp homologues of chromosome 3 (Online Resource S1). In addition, we expected zero to one small 5S rDNA locus in a distal region of unrecognized Fp chromosomes. All changes from each generation studied, in *F. pratensis* × *L. perenne* hybrids with equal number of *Festuca* and *Lolium* chromosomes, compared with the expected number and position of rDNA sites, were treated as possible variations in the rDNA loci pattern.

### Chromosome preparations

Root tips of all 30 F_2_-F_4_ plants were collected in ice water, refrigerated for 24 h, fixed in ethanol with glacial acetic acid (3:1, *v*/*v*), and then stored at −20 °C until use. Further treatment was performed according to Zwierzykowski et al. (1998). Chromosome analysis was carried out on 3-5 well-spread metaphases. Each chromosomal preparation was derived from a different single root tip, so that each preparation corresponded to one individual.

### DNA probes

Three kinds of probes were used: (i) total genomic DNA from *F. pratensis* and *L. perenne* extracted from young leaves using Novabeads Plant DNA Maxi Kit according to the manufacturer’s procedure (Novazym Poland; after modifications), and further treatment of extracted DNA was carried out as described by Książczyk et al. (2010); (ii) the 5S rDNA probe was generated by PCR amplification of a 410-bp *Bam*HI sub-clone of the 5S rDNA from the wheat clone pTa794 (Gerlach and Dyer 1980) and also labeled by PCR with tetramethyl-rhodamine-5-dUTP (Roche) by using universal M13 “forward” (5′-CAG GGT TTT CCC AGT CAC GA-3′) and “reverse” (5′-CGG ATA ACA ATT TCA CAC AGG A-3′) sequencing primers. The thermal cycling program was as follows: 94 °C for 1 min, 39 cycles of 94 °C for 40 s, 55 °C for 40 s, and 72 °C for 90 s, and finally, 72 °C for 5 min; (iii) the 26S rDNA probe, used for detection of 35S rDNA loci, was made by nick translation of a 2.3-kb *Cla*I sub-clone of the 26S rDNA coding region of *Arabidopsis thaliana* (Unfried and Gruendler 1990) with digoxigenin-11-dUTP (Roche). The conditions for this reaction were as follows: 15 °C for 95 min and 65 °C for 10 min.

### In situ hybridization (FISH and GISH)

The FISH procedure was performed as described by Książczyk et al. (2010). The Lp and Fp chromosomes identified by rDNA-FISH were numbered according to Thomas (1981). For distinguishing the two subgenomes of the hybrids, GISH was done using the total genomic DNA of Lp and Fp as a probe and block, respectively. Before GISH, incubation of slides previously subjected to FISH experiments was carried out as described by Książczyk et al. (2010). The GISH procedure was adapted from Kosmala et al. (2006), with minor modifications (Książczyk et al. 2010). The following observations were made for each plant studied: (i) the total number of complete Lp and Fp rDNA-bearing chromosomes, (ii) the total number of complete Lp and Fp non-rDNA-bearing chromosomes, (iii) the total number of recombinant Lp and Fp rDNA-bearing chromosomes, (iv) the total number of recombinant Lp and Fp non-rDNA-bearing chromosomes, (v) the total number of complete and recombinant Lp and Fp chromosomal arms with rDNA site, (vi) the total number of complete and recombinant Lp and Fp chromosomal arms without rDNA site, and (vii) frequency of rDNA-bearing chromosomes (3L as well as 2F and 3F) involved in recombination.

### Image capturing and processing

All images were acquired using either an Olympus XM10 CCD camera attached to an Olympus BX 61 automatic epifluorescence microscope, or an F-View II CCD camera attached to an Olympus BX 60 epifluorescence microscope. Image processing and superimpositions were done using Olympus Cell-F imaging software and Micrographx Picture Publisher software.

### Statistical analysis

To evaluate the influence of three generations on Lp and Fp chromosome changes, and to study difference between both genomes, cytogenetic data were statistically processed by the Pearson’s chi-squared test at *P* ≤ 0.05, according to standard procedures within GenStat® version 15.1 (Payne et al. 2012).

## Results

### rDNA loci pattern versus recombination in the F_2_-F_4_ generations

Among plants of the three generations the number of 5S rDNA sites ranged from 4 to 6, with a predominant number of five signals, although six signals of 5S rDNA loci were observed only in plants of the F_3_ generation (Table 1). There were two to three sites in Lp of the F_2_, one to three in Lp of the F_3_, and two to four in Lp of the F_4_ (chromosome no. 3). In addition, there were one to two sites in Fp (chromosome no. 3; F_2_ and F_3_) and zero to two sites in Fp (chromosome no. 3; F_4_) large (main) 5S rDNA sites interstitially located, while a small 5S rDNA locus was found in a distal region of one (F_2_ and F_4_) or two (F_3_) unrecognized Fp chromosomes. In one F_2_ plant, the additional small 5S rDNA locus was distally located in the recombinant undifferentiated Lp chromosome (data not presented), but the recombinant Lp chromosome was apparently lost in later generations. The number of 35S rDNA sites ranged from 8 to 11 in the F_2_, 8 to 10 in the F_3_, and 7 to 12 in the F_4_ (Table 1). The number of clearly identifiable Lp homologues of chromosome 3 ranged 2–3 (F_2_), 1–3 (F_3_), and 2–4 (F_4_), although plants with both Lp homologues of chromosome 3 were “dominant” over the generations (24/30 plants). Through the generations studied, 4 to 7 (F_2_), 3 to 6 (F_3_), 1 and 4 to 6 (F_4_) signals of 35S rDNA sites were always located at secondary constrictions on Lp homologues of cytologically undifferentiated chromosomes 1 and 2, as well as proximally located close to the centromere of chromosome 7. Signals of 35S rDNA sites were also located at the secondary constriction on Lp and Fp homologues of chromosomes 3 and 2, respectively, showing the differentiation in their patterns as follows: 2–3 Lp and 1–2 Fp (F_2_), 1–3 Lp, and 2 Fp (F_3_), and also 2–4 Lp and 1–4 Fp (F_4_).

**Table 1:** Number and chromosomal position of rDNA sites in plants of F_2_-F_4_ generations derived from the allotetraploid *F. pratensis* × *L. perenne* hybrid

Generation/plant no.	2*n*	Chromosome ratio Lp/Fp	No. of rDNA sites (position^a^)					No. of chromosomes with both rDNA sites
			5S rDNA			35S rDNA		
			Lp (is)	Fp (is)	Fp^b^ (d)	Lp (sc/p)	Fp (sc/p)	
F2-7	28	14:14	2	2	1	6	2	2
F2-9	28	14:14	2	2	1	7	2	2
F2-13	28	14:14	2	2	0	7	2	2
F2-15	28	14:14	2	2	1	6	2	2
F2-80	28	14:14	2	2	1	9	2	2
F2-126	28	14:14	2	2	0	9	2	2
F2-11	28	15:13	2	2	1	8	2	2
F2-28	28	15:13	2	2	1	8	2	2
F2-122	28	15:13	3	1	1	7	2	3
F2-79	28	17:11	2	2	1	9	1	2
F3-28	28	14:14	2	2	2	8	2	2
F3-34	28	14:14	2	2	0	8	2	2
F3-150	28	14:14	2	2	0	7	2	2
F3-18	28	13:15	2	2	2	6	2	2
F3-57	28	13:15	2	2	1	8	2	2
F3-123	28	13:15	2	2	0	7	2	2
F3-1	28	15:13	2	2	1	6	2	2
F3-96	28	15:13	3	1	1	6	2	3
F3-106	28	16:12	1	2	1	7	2	1
F3-139	28	16:12	2	2	0	7	2	2
F4-28	28	14:14	2	2	1	8	2	2
F4-33	28	14:14	2	2	0	8	2	2
F4-53	28	14:14	2	2	1	8	1	2
F4-83	28	14:14	2	2	0	8	2	2
F4-6	28	12:16	2	2	0	3	4	2
F4-10	28	15:13	2	2	0	6	3	2
F4-104	28	15:13	2	2	1	6	3	2
F4-109	28	16:12	3	1	1	8	2	3
F4-135	28	17:11	3	1	1	9	2	3
F4-25	28	18:10	4	0	0	9	3	4
Test result^c^		n.s.	n.s.	n.s.	n.s.	n.s.	*P* = 0.022	n.s.

*Lp L. perenne* chromosomes, *Fp F. pratensis* chromosomes

^a^Position of rDNA sequences is shown in brackets: interstitial (is), distal (d), secondary constriction (sc), proximal (p)

^b^Unrecognized *F. pratensis* chromosome with an additional 5S rDNA locus

^c^The distribution of values for 5S and 35S rDNA loci between generations was compared, and significant differences between distributions for Lp and Fp were assessed using Pearson’s chi-squared test (*P* ≤ 0.05); statistically significant difference for Fp genome-like 35S rDNA between generations was found at *P* = 0.022 (n.s. means *P* > 0.05).

Generally, 30 F_2_-F_4_ plants showed up to 11 various rDNA loci patterns, of which 6 patterns were repeated two to six times (Table 1). Thirteen F_2_-F_4_ plants had an equal number of chromosomes, 14Lp and 14Fp, showing up to eight various rDNA loci patterns, of which four patterns were repeated two to three times (Table 1). The hypothetical model of F_1_ Fp × Lp karyotype (14Lp:14Fp) (Online Resource S1) assumes a presence of four signals (2Lp + 2Fp) of 5S rDNA and nine signals (7Lp + 2Fp) of 35S rDNA, which was only observed in one plant of the F_3_ and F_4_ and in two plants of the F_2_. However, the expected four to five signals of 5S rDNA and seven to nine signals of 35S rDNA were observed in 6 out of 13 plants (14Lp:14Fp) of the F_2_-F_4_ generations. In seven remaining plants, six had the expected number of 5S rDNA loci (four to five), but an unexpected number of 35S rDNA (10–11); the seventh plant had six unexpected signals of 5S rDNA and ten signals of 35S rDNA (Table 1). In two out of four Fp × Lp plants with a lower number of Lp chromosomes (12–13 Lp chromosomes; Table 1), the hypothetical (5S/35S: 4/9) and expected (4/7) number of rDNA loci was observed (13Lp:15Fp and 12Lp:16Fp), while the other two plants (13Lp:15Fp) had the expected number of 5S or 35S rDNA loci. Among 13 Fp × Lp plants with the higher number of Lp chromosomes (15–18 Lp chromosomes; Table 1), the hypothetical (4/9) and expected (4/9, 5/8, 5/9) number of rDNA loci was observed in seven plants (five plants with 15Lp:13Fp and two plants with 16Lp:12Fp; Table 1), although the distribution of rDNA loci was consistent with the hypothetical model of rDNA loci pattern only in one plant (16Lp:12Fp) (Online Resource S1). Six other plants had unexpected 5S and 35S rDNA loci patterns. It is worth mentioning that 4/12 (5S/35S) rDNA loci pattern occurred in the Fp × Lp plant with 18Lp and 10Fp chromosomes (Table 1), and the number and position of rDNA loci were also not consistent with the hypothetical model of rDNA loci pattern (Online Resource S1), showing three homologues of Fp chromosome 2 (instead of two) and a lack of two homologues of Fp chromosome 3, which might be absent due to a lower number of Fp chromosomes in this plant (Table 1).

**Fig. 1:**
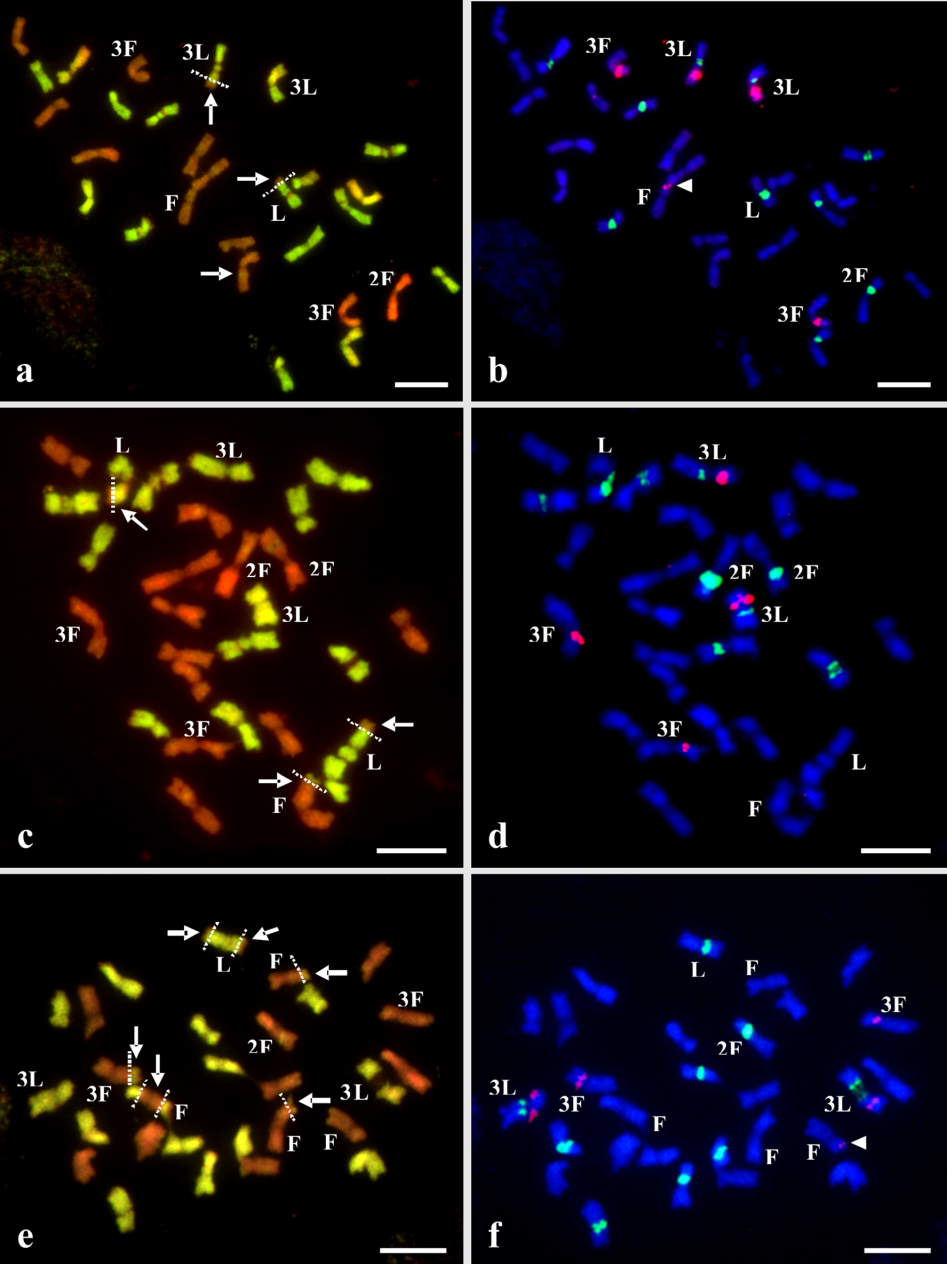
Chromosome identification of parental species in plants of the F_2_-F_4_ generations derived from *F. pratensis* (4*x*) × *L. perenne* (4*x*) hybrid using GISH (**a**, **c**, **e**) and FISH (**b**, **d**, **f**). GISH images (**a**, **c**, **e**) were created after FISH hybridization using total genomic DNA from Lp as a probe labeled with digoxigenin and detected by anti-digoxigenin conjugated with fluorescein (*green*/*yellow*), with blocking genomic DNA of Fp (*orange*/*red*); chromosomes were counterstained with propidium iodide. FISH images (**b**, **d**, **f**) were created using probes as follows: (i) 5S rDNA labeled with rhodamine (*red*) and (ii) 26S rDNA labeled with digoxigenin and detected by anti-digoxigenin conjugated with FITC (*green*); chromosomes were counterstained with DAPI (*blue*). GISH and FISH images are marked by *white arrows* indicating Lp and Fp recombinant chromosomes (R), by *white arrowheads* indicating additional location of 5S rDNA locus, and by the *white lines* with intervals indicating recombination breakpoints. **a**, **b** F_2_ plant [17Lp (2R) +11Fp (1R)]. **c**, **d** F_3_ plant [14Lp (2R) +14Fp (1R)]. **e**, **f** F_4_ plant [14Lp (1R) +14Fp (4R)]. The nomenclature of rDNA-bearing chromosomes (*Arabic numerals*) follows the system of Thomas (1981). *Uppercase letters* denote the genomic origin of tagged chromosomes. *Scale bars* represent 5 μm

**Fig. 2:**
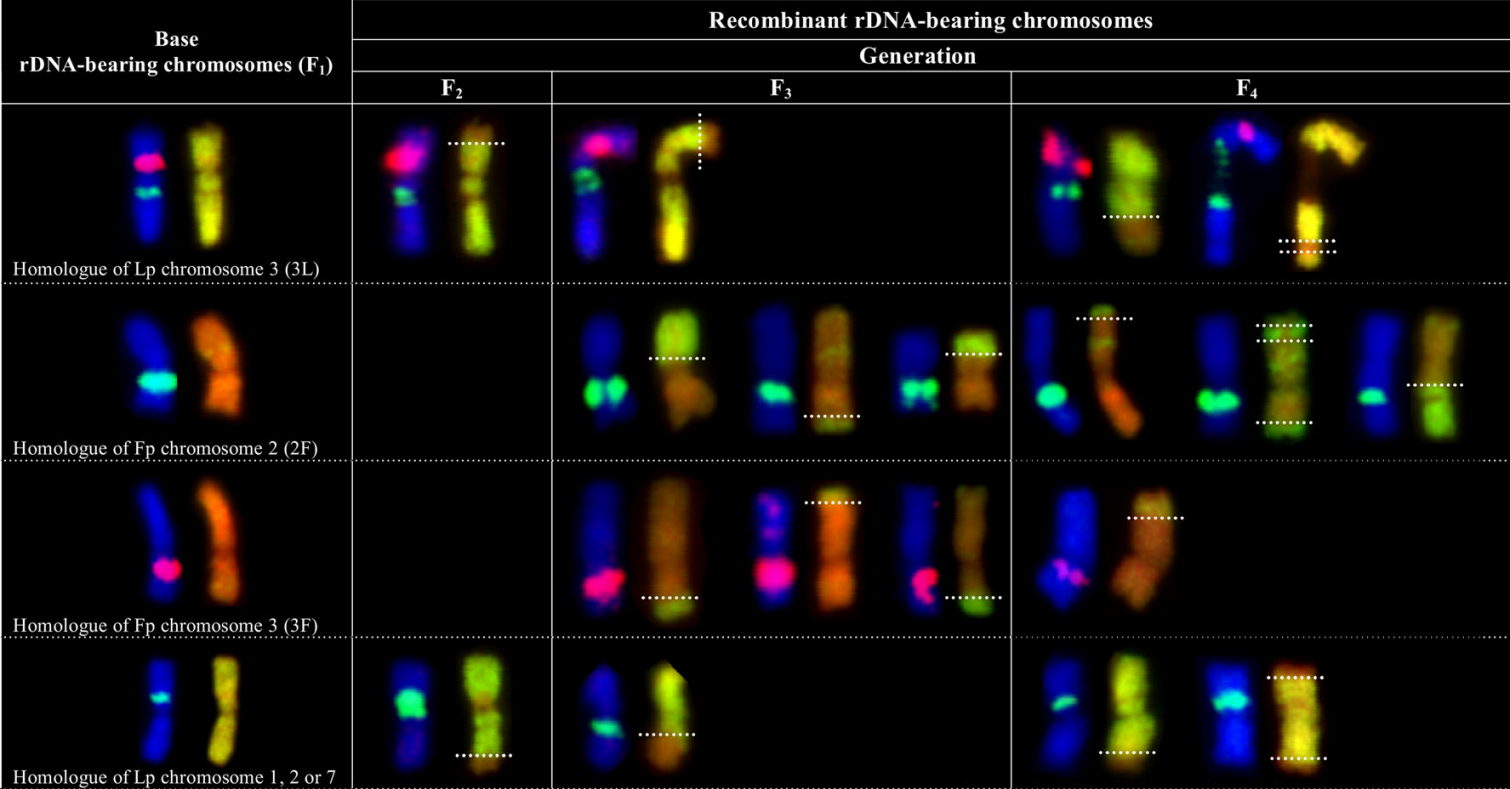
rDNA-FISH/GISH of known Lp and Fp rDNA-bearing chromosomes in plants of the F_2_-F_4_ generations derived from *F. pratensis* (4*x*) × *L. perenne* (4*x*) hybrid. Data of FISH/GISH analyses of base rDNA-bearing chromosomes from the F_1_ generation were published previously (Książczyk et al. 2010). The color of the chromosome band label indicates the fluorochrome used in each experiment (*pink* for rhodamine; 5S rDNA, *green* and *green*/*yellow* for FITC; 35S rDNA and Lp genomic DNA, respectively). FISH painted chromosomes were counterstained with DAPI (*blue*), while GISH ones were counterstained with propidium iodide (*red*)

Sixty-five Lp homologues of chromosome 3 were observed in Fp × Lp plants (Table 1), and 48 of these chromosomes did not undergo numerical changes, while among 157 Lp homologues of chromosomes 1, 2, and 7, only 16 Lp rDNA-carrying ones were stable, and their number was consistent with the hypothetical model (Online Resource S1). In the F_2_-F_4_ plants studied, among 63 homologues of Fp chromosome 2, and 54 homologues of Fp chromosome 3, no numerical changes were noted in 48 and 50 homologues, respectively. In the F_2_ generation, Lp homologue(s) of chromosome 3 was rarely involved in recombination, showing rearrangement (only one case; Figs. 1a, b and 2), while no Fp homologues of chromosomes 2 and 3 were found to be rearranged. In the F_3_ generation, the Lp homologue of chromosome 3 and Fp homologues of chromosomes 2 and 3 were involved in recombination, showing rearrangements (14 cases; Fig. 2), and, in turn, in the F_4_ generation, the Lp homologue of chromosome 3 and Fp homologues of chromosomes 2 and 3 (Fig. 1e, f) were also recombined (21 cases; Fig. 2). Over the generations, the variation in the number of rDNA-carrying chromosomes of both parental genomes seemed to be asymmetrical and genome-dependent (Table 1); statistically significant difference for rDNA-bearing chromosomes between generations was found for Fp chromosome 2 (35S rDNA) at *P* = 0.022.

### Structural dynamics of rDNA loci patterns in the F_2_-F_4_ generations

The frequency profile of complete and recombinant rDNA-bearing and non-rDNA-bearing chromosomes of both genomes in the hybrids of the three generations is given in Online Resource S2. Recombinant rDNA-bearing chromosomes were only observed in the Lp genome of the F_2_ (Figs. 1a, b and 2) (mean 0.8/genotype), in both Lp (mean 0.9) and Fp (mean 1.1) of the F_3_, as well as in both Lp (mean 0.9) and Fp (mean 1.8) of the F_4_ (data not presented). Over the generations, 26 (F_2_), 47 (F_3_), and 69 (F_4_) recombined Lp and Fp chromosomes were observed (Table 2). Among 26 recombined chromosomes in the F_2_ generation, 6 Lp and 12 Fp were non-rDNA-bearing ones. No Fp, but eight Lp were rDNA-bearing (Table 2), of which one Lp had both 5S and 35S rDNA loci (chromosomes 3, Figs. 1a, b and 2), six had Lp 35S rDNA locus (chromosomes 1, 2, or 7, Figs. 1a, b and 2), and one unknown Lp had 5S rDNA locus (data not showed). Among 47 recombined chromosomes of the F_3_, 9 Lp and 18 Fp were non-rDNA-bearing ones, while 9 Lp and 11 Fp were rDNA-bearing, of which 4 Lp had both 5S and 35S rDNA loci (chromosome 3, Fig. 2), 5 had Lp 35S rDNA locus (chromosomes 1, 2, or 7, Figs. 1c, d and 2), 6 had Fp 35S rDNA locus (chromosome 2), 4 had Fp large 5S rDNA locus (chromosome 3), and 1 had Fp small 5S rDNA locus (unknown chromosome). It should be pointed out that three types of Fp rDNA-bearing chromosomes (two known and one unrecognized; Table 1) were found to be more frequently recombined (11 cases) based on five known types of Lp rDNA-bearing ones (nine cases). Among 69 recombined chromosomes in the F_4_, 12 Lp and 30 Fp were non-rDNA-bearing ones, 9 Lp and 18 Fp were rDNA-bearing ones, of which 5 Lp had both 5S and 35S rDNA loci (chromosome 3, Figs. 1e, f and 2), 4 had Lp 35S rDNA locus (chromosomes 1, 2, or 7, Fig. 1e, f), 11 had Fp 35S rDNA locus (chromosome 2), 6 had Fp large 5S rDNA locus (chromosome 3, Fig. 1e, f), and 1 had Fp small 5S rDNA locus (unknown chromosome, Fig. 1e, f). Again, Fp rDNA-bearing chromosomes were found to be the most frequently recombined in the F_4_. It is worth mentioning that the number of recombined rDNA-bearing chromosomes was doubled for Fp ones, comparing both genomes (Table 2, Online Resource S2). Over the generations, the variation in the number of recombinant Lp and Fp arms with (m+) and without (m−) rDNA loci also seemed to be genome-dependent (Table 2).

**Table 2:** Number of non-recombined and recombined non-rDNA- and rDNA-bearing chromosomes and their genome assignment in plants of F_2_-F_4_ generations derived from the allotetraploid *F. pratensis* × *L. perenne* hybrid

Generation/plant no.	Chromosome ratio M+/M−	No. of non-recombined chromosomes				No. of recombined chromosomes				No. of recombined arms			
		M+		M−		M+		M−		m+		m−	
		Lp	Fp	Lp	Fp	Lp	Fp	Lp	Fp	Lp	Fp	Lp	Fp
F2-7	11:17	6	5	7	8	0	0	1	1	0	0	0	0
F2-9	12:16	6	5	6	7	1	0	1	2	1	0	0	0
F2-13	11:17	7	4	5	10	0	0	2	0	0	0	0	0
F2-15	11:17	6	5	7	8	0	0	1	1	0	0	0	0
F2-80	14:14	9	5	5	9	0	0	0	0	0	0	0	0
F2-126	13:15	9	4	5	9	0	0	0	1	0	0	0	0
F2-11	13:15	6	5	7	7	2	0	0	1	0	0	2	0
F2-28	13:15	6	5	7	6	2	0	0	2	2	0	0	0
F2-122	11:17	6	4	7	7	1	0	1	3	1	0	0	0
F2-79	13:15	7	4	8	6	2	0	0	1	1	0	1	0
F3-28	14:14	7	6	6	5	1	0	0	3	1	0	0	0
F3-34	12:16	7	3	5	7	1	1	1	3	1	0	0	1
F3-150	11:17	6	4	6	9	1	0	1	1	1	0	0	0
F3-18	12:16	6	6	6	8	0	0	1	1	0	0	0	0
F3-57	13:15	6	5	5	8	2	0	0	2	2	0	0	0
F3-123	11:17	6	1	7	8	1	3	0	2	1	1	0	2
F3-1	11:17	4	4	8	7	2	1	1	1	2	0	0	1
F3-96	11:17	6	3	8	6	0	1	1	3	0	0	0	1
F3-106	12:16	7	3	6	6	0	2	3	1	0	1	0	1
F3-139	11:17	6	1	8	8	1	3	1	1	1	1	0	2
F4-28	13:15	8	4	4	5	0	1	2	4	0	0	0	1
F4-33	12:16	6	2	6	7	2	2	0	3	2	2	0	0
F4-53	12:16	7	3	5	7	1	1	1	3	1	0	0	1
F4-83	12:16	6	4	6	4	2	0	0	6	1	0	1	0
F4-6	9:19	2	1	9	9	1	5	0	1	1	1	0	4
F4-10	10:18	6	4	8	6	0	1	1	2	0	1	0	0
F4-104	12:16	6	3	7	6	0	3	2	1	0	0	0	3
F4-109	12:16	7	2	7	5	1	2	1	3	1	1	0	1
F4-135	13:15	7	2	7	5	2	2	1	2	1	0	1	2
F4-25	12:16	9	2	5	2	0	1	4	5	0	0	0	1
Test result^a^	n.s.	n.s.	*P* = 0.001	n.s.	n.s.	n.s.	*P* = 0.001	n.s.	n.s.	n.s.	n.s.	n.s.	*P* = 0.013
Test result^b^	n.a.	n.a.		n.a.		n.s.		*P* = 0.008		*P* = 0.035		n.s.	

*Lp L. perenne* chromosomes, *Fp F. pratensis* chromosomes, *M+* Lp and Fp marked chromosomes, *M−* Lp and Fp non-marked chromosomes, *m+* Lp and Fp chromosomal arms with rDNA locus, *m−* Lp and Fp chromosomal arms without rDNA locus, *n.a.* means not analyzed

*n.s. P* > 0.05

^a^The distribution of M+/m+ and M−/m− values for non-recombined and recombined chromosomes as well as recombined arms between generations was compared, and significant differences between distributions for Lp and Fp were assessed using Pearson’s chi-squared test (*P* ≤ 0.05); statistically significant differences between generations were found both for Fp rDNA-carrying non-recombined and recombined chromosomes (at *P* = 0.001) and for Fp non-rDNA-carrying recombined arms (at *P* = 0.013)

^b^The distribution of M+/m+ and M−/m− values for recombined chromosomes and arms between both genomes was compared, and significant differences between distributions for Lp and Fp were assessed using Pearson’s chi-squared test (*P* ≤ 0.05); statistically significant differences between Lp and Fp genomes were found with respect to the number of recombined chromosomes without marker (M−) (at *P* = 0.008), and also with respect to the number of recombined arms with marker (m+) (at *P* = 0.035)

The distribution of values for rDNA- (M+) and non-rDNA-bearing (M−) chromosomes between generations was compared (Table 2), and statistically significant structural differences for rDNA-bearing chromosomes between generations were found for non-recombined (*P* = 0.001) and recombined (*P* = 0.001) Fp rDNA-bearing ones, and also for Fp non-rDNA-bearing arms of recombined chromosomes with the marker (*P* = 0.013). In the case of the remaining structural characters given in Table 2, no statistically significant differences for Lp and Fp chromosomes between generations were found at the *P* < 0.05. The distribution of M+/m+ and M−/m− values for recombined chromosomes and arms between both genomes was also compared (Table 2 and Online Resource S3), and statistically significant differences between distributions for Lp and Fp genomes were found for *P* = 0.008 with respect to the number of recombined chromosomes without marker (M−), and also for *P* = 0.035 with respect to the number of recombined arms with marker (m+).

## Discussion

Our results show a *Festuca*-like and *Lolium*-like dynamic pattern of chromosome variation in the *F. pratensis* × *L. perenne* hybrids, occurring during early, F_2_-F_4_, generations following hybridization. The numerous changes, which seemed to occur independently within the Fp and Lp genomes, often altered non-3L, non-2F, and non-3F chromosomes. The presence of rearrangements in rDNA-bearing chromosomes concerns chromosomal arms with or without rDNA loci. The comparison of rDNA profiles in plants observed in the F_1_, and then in F_2_-F_4_ generations revealed, as it was expected, further differentiation in number and position of rDNA loci and parental split of rDNA loci patterns in Fp × Lp hybrids.

Over the F_2_-F_4_, the proportion of rearranged rDNA-bearing and non-rDNA-bearing chromosomes of parental genomes increased from generation to generation, and the frequency was higher for non-marked chromosomes than for rDNA-carrying ones. The number of recombinant and non-recombinant Lp and Fp rDNA-bearing chromosomes, and the frequency of structural rearrangements in rDNA-bearing ones, also increased from generation to generation, although the respective value of these characters was always higher for Fp chromosomes with rDNAs. The hypothesis was that over the generations the variation of parental genomes is asymmetrical, as borne out in the present work, and significant differences in this variation were always biased in favor of the Fp chromosomes. The recombination pattern was consistent with previous observations recorded for Fp chromosomes, in which there was an increase in the recombination profile in plants from the F_2_ to the F_8_ generation in selected population of Fp × Lp hybrids (Zwierzykowski et al. 2006, 2011). The present work shows that the non-recombinant and recombinant Lp rDNA-bearing chromosomes remained on a comparable level over the generations, while the non-recombinant and recombinant Fp rDNA-bearing ones were much increased, and exceeded double the Lp value for Fp in the F_4_. This observation does not suggest, however, a greater capacity of the Lp genome to be structurally more stable than the Fp one, but the profile of Fp chromosomes to recombine more often than those of Lp ones has been already proved to some extent (Zwierzykowski et al. 2006, 2011). In the F_2_-F_4_ generations of the hybrids, we observed an asymmetrical pattern of rDNA-carrying chromosome variation in the number of recombinant Lp and Fp arms with and without rDNA loci. In the Lp and Fp recombination pattern of arms with and without rDNA locus, the distribution of values increased over the generations, but statistically significant difference between generations was found in the Fp genome for arms without rDNA locus. This indicates that in the case of marked chromosomes, the Fp genome was more affected by changes in arms without any rDNA locus. On the other hand, it is showed that statistically significant differences between Lp and Fp genomes were found in respect to the number of recombined chromosomes without rDNA locus (M−) and recombined arms with rDNA one (m+) (Online Resource S3b, c). This means that in the case of the number of recombined M− chromosomes, no recombination event is more frequent for Lp chromosomes, but two or three recombination events are more frequent for Fp ones (Online Resource S3b), although no statistically significant differences were found between distribution of these characters over the generations separately for Lp and Fp genomes. Similarly, no statistically significant differences were found between distribution of m+ character over the generations separately for Lp and Fp genomes, but the comparison of this character between Lp and Fp genomes showed significant difference; no recombination event is more frequent for Fp m+ chromosomes, but one or two recombination events are more frequent for Lp m+ ones (Online Resource S3c). This confirms our observations found in present work that statistically significant difference in the recombination profile was found for Fp-genome-like m− arms. On the contrary, statistically significant differences between distributions of M+ character were found over the generations separately for Lp and Fp genomes, but no difference was found between distributions of this character for Lp and Fp genomes, when compared. Again, such a general tendency of recombination profiles in Lp and Fp chromosomes seems to be in agreement with the previous data found in plants of the F_2_-F_4_ generations (Zwierzykowski et al. 2012), confirming a balance of chromatin, which progressively to favors the dominant *Lolium* genome and higher predisposition of *Festuca* chromosomes to be structurally more often modified (Zwierzykowski et al. 2006).

Recombination of chromosomes with arms carrying rDNA loci has also been found in wheat/rye translocations (Lukaszewski and Gustafson 1983), in *Triticum* × *Dasypyrum* hybrids (Minelli et al. 2005) and in allotetraploid *Secale* × *Dasypyrum* forms (Książczyk et al. 2011b). A similar approach using FISH/GISH has already been used in F_1_ plants of *F. pratensis* × *L. perenne* hybrids (Książczyk et al. 2010), in which existing cytological landmarks showed some vulnerability of particular Lp and Fp rDNA loci to change their position, especially when those sequences were located at a secondary constriction, which is relatively unstable chromosomal region (Schubert and Wobus 1985). Significantly, rDNAs may be targets of rearrangements, as was shown in newly synthesized allotetraploids of *Brassica* species (Książczyk et al. 2011a; Xiong et al. 2011). Chromosomal rearrangements may involve many processes, e.g., activation of transposable elements or epigenetic regulation (Soltis and Soltis 1993), as well as structural rearrangements such as inversions and translocations (Levin 2004). The variation in the number and location of 35S rDNA signals found in *L. perenne* can be due to the formation of breaks and/or gaps in 35S rDNA sites and can randomly fragment the 35S rDNA regions (Huang et al. 2008; Rocha et al. 2014). The incidence of fragile sites may be involved in the process of chromosomal variation, including rearrangements and amplifications constituting a potential mechanism for speciation (Brown and O’Neill 2010) and its role in evolution, by asserting that fragile sites may generate chromosomal instability as representing fragile regions of the genome and are able to undergo recombination events (Ruiz-Herrera and Robinson 2007). Thus, the variation of rDNAs has led to the hypothesis that rDNA clusters are mobile (Schubert and Wobus 1985) and that some rDNA changes in chromosomal location may be activated by transposons enabling the traveling of (r)DNA to a new site (Raskina et al. 2004). A transposase-mediated transposition of rDNA might be postulated as the key mechanism in chromosome evolution (Raskina et al. 2004; Datson and Murray 2006; Pedrosa-Harand et al. 2006), and such a model could be responsible for the presence of distally located new loci of 5S rDNA within *F. pratensis* cultivars (Książczyk et al. 2010). It has also been found by many authors that retroelements play a major role in shaping and remodeling genomes during evolution by their influence on chromosome stability (Feuillet and Keller 2002). Langdon et al. (2000) have shown that a single ancestral family of retrotransposons related to the Ty3-gypsy family is the source of all Poaceae centromere-specific retroelement sequences. In solanaceous species, maize, rice and *Arabidopsis*, terminal-repeat retrotransposons *in miniature* (TRIM), the smallest known LTR retrotransposons, can be mobilized by other retroelements and are found to be actively involved in the reshaping of their genomes (Witte et al. 2001). It is anticipated that TRIM-like elements might exist in forage grasses, such as *L. perenne* and *F. pratensis*, and could be involved in some rDNA mobility. In the Fp genome, the main 5S rDNA loci are closely embedded in pericentromeric heterochromatin that is typically rich in transposable elements, so a transposon-mediated rearrangement could contribute to a loss or transposition of 5S rDNA sequences observed in Fp-genome-like chromosomes of Fp × Lp hybrids, suggesting extensive chromosome rearrangements resulting from genome imbalance during polyploid formation, as it was recently shown in *Tragopogon* allotetraploids (Malinska et al. 2010). The question is whether a similar model of (5S) rDNA mobility is present in the tetraploid *F. pratensis* × *L. perenne* hybrids? Thus, various proportions of centromere-specific retroelements between the two syntenic 3F and 3L chromosomes carrying rDNA loci in the proximal region might account for differences found in the recombination of arms with and without rDNA loci between both genomes.

Changes of rDNA sites, e.g., gaining of 5S ribosomal RNA (rRNA) genes, appears to have occurred more frequently in four tetraploid F_1_ plants of *F. pratensis* × *L. perenne* hybrid (Książczyk et al. 2010), being used to obtain the F_2_ generation. In the present work, the Fp genome-like 5S rDNA loci found in unrecognized chromosomes were more affected by numerical changes than the Fp and Lp genome-like 5S rDNA ones found in homologues of chromosome 3. This postulates the existence of genome-dependent dynamics of 5S rDNA loci pattern. In the majority of studied Fp × Lp hybrids, the amplification of 5S rRNA gene loci in Fp genome-like chromosomes was observed, and such additional and near-terminally located 5S rDNA sites were also found in F_1_ plants previously studied (Książczyk et al. 2010). The origin and uniparental extent of 5S rDNA variation remains unclear, although in some plants, “novel” or migrated loci were positioned at near-terminal and terminal regions of the chromosomes (Li and Zhang 2002), suggesting that the loci might change position through dispersion of minor loci without chromosome rearrangements (Dubcovsky and Dvorák 1995). We think that this mechanism could explain the appearance of novel 5S rDNA sites in Fp chromosomes. Interchromosomal exchanges might be facilitated by the terminal or near-terminal location of the rDNA loci, and the terminal location of the rDNA locus and the loss of its interstitial site suggests that concerted evolution of the particular rDNA locus (and sequence homogeneity) has probably occurred (Li and Zhang 2002), and inter-locus unequal crossing over could be proposed to play a role in concerted evolution (Wendel et al. 1995; Raskina et al. 2004; Pedrosa-Harand et al. 2006). The most intriguing aspect in the 5S rDNA variation observed in Fp-genome-like chromosomes in the hybrids is the appearance of novel 5S rDNA loci, which may result from transposon activity and may not be associated with the loss of major 5S rDNA sites on Fp homologues of chromosome 3 (the loss of 5S rDNA site resulted rather from an incomplete number of Fp chromosomes in some plants of the F_2_-F_4_ generations), and it is likely that the Fp genome does not address this hypothesis in a similar way as proposed for concerted evolution in other plants. Further analyses are necessary to prove or reject this hypothesis as well as for deeper understanding of the mechanisms responsible for the genome-dependent rDNA dynamics in genomes of the *Festuca*-*Lolium* complex.

## Conclusions

Our results show that the chromosome variation in plants of the F_2_-F_4_ generations derived from the F_1_ hybrid of *F. pratensis* × *L. perenne* seem to argue for genome-dependent dynamics of chromosome changes. They also show the independent character of rDNA loci patterns within *L. perenne* and *F. pratensis* genomes. A statistically significant difference between distributions of values for 35S rDNA loci over the generation was found for *F. pratensis* genome-like chromosome 2, and *F. pratensis* genome-like chromosomes were more affected by rDNA loci changes, showing the presence of an additional 5S rDNA locus found in unrecognized *F. pratensis* chromosomes. Moreover, statistically significant differences between parental genomes were found both for non-recombined and recombined *F. pratensis* rDNA-bearing chromosomes (M+) and for recombined *F. pratensis* non-rDNA-carrying arms (m−) of marked chromosomes. Statistically significant differences between *L. perenne* and *F. pratensis* genomes were also found for recombined *F. pratensis* chromosomes without marker (M−) and recombined *F. pratensis* chromosomal arms of marked chromosomes (m+), indicating a tendency of *F. pratensis* genome-like chromosomes to be less stable in plants of the F_2_-F_4_ generations.

## Electronic supplementary material

Below is the link to the electronic supplementary material.

ESM 1 (DOC 44 kb)

Online Resource S1 Ideograms of the rDNA loci number and position in *L. perenne* (A), *F. pratensis* (B) and a hypothetical model of F_1_ karyotypes illustrating rDNA-bearing chromosome complement in *F. pratensis* (4*x*) × *L. perenne* (4*x*) hybrid (C). The rDNA loci pattern of *L. perenne* and *F. pratensis* is taken from Książczyk et al. (2010). (PDF 103 kb)

Online Resource S2 Frequency profile of complete and recombinant *L. perenne* and *F. pratensis* rDNA-bearing and non-rDNA-bearing chromosomes in plants of the F_2_-F_4_ generations derived from *F. pratensis* (4*x*) × *L. perenne* (4*x*) hybrid. M+ Lp and Fp rDNA-bearing chromosomes, M- Lp and Fp non-rDNA-bearing chromosomes, M+(L) Lp and M+(F) Fp rDNA-bearing chromosomes, M-(L) Lp and M-(F) Fp non-rDNA-bearing chromosomes, M+(L)R recombinant Lp rDNA-bearing chromosomes, M+(F)R recombinant Fp rDNA-bearing chromosomes, M+(L + F)R total number of recombinant Lp and Fp rDNA-bearing chromosomes, M-(L + F)R total number of recombinant Lp and Fp non-rDNA-bearing chromosomes. (PDF 51 kb)
