# Supplementary material for: Genome-dependent chromosome dynamics in three successive generations of the allotetraploid Festuca pratensis × Lolium perenne hybrid
Source: Protoplasma. 2014 Dec 6;252(4):985–96. doi: 10.1007/s00709-014-0734-9 (PMC4491343; doi:10.1007/s00709-014-0734-9)
Supplement: Supplementary file 1 — (DOC 44 kb) [file 709_2014_734_MOESM1_ESM.doc]

**Table S3a.** Distribution of values in plants of F_2_-F_4_ generations with respect to the number of recombined *L*. *perenne* and *F*. *pratensis* chromosomes with marker (M+)

| Type of chromosome recombination | Number of recombined chromosomes | | | | |
| --- | --- | --- | --- | --- | --- |
|  | 0 | 1 | 2 | 3 | 5 |
| Recombined M+ *L*. *perenne* | 12 | 10 | 8 | 0 | 0 |
| Recombined M+ *F*. *pratensis* | 15 | 7 | 4 | 3 | 1 |

Pearson’s chi-squared 6.20. Probability 0.162. No difference between distributions for *L*. *perenne* and *F*. *pratensis* chromosomes.

**Table S3b.** Distribution of values in plants of F_2_-F_4_ generations with respect to the number of recombined *L*. *perenne* and *F*. *pratensis* chromosomes without marker (M-)

| Type of chromosome recombination | Number of recombined chromosomes | | | | | | |
| --- | --- | --- | --- | --- | --- | --- | --- |
|  | 0 | 1 | 2 | 3 | 4 | 5 | 6 |
| Recombined M- *L*. *perenne* | 11 | 14 | 3 | 1 | 1 | 0 | 0 |
| Recombined M- *F*. *pratensis* | 2 | 12 | 6 | 7 | 1 | 1 | 1 |

Pearson’s chi-squared 13.88. Probability 0.008. Significant difference between distributions; ‘0’-case of recombination is more frequent for *L*. *perenne* chromosomes, while ‘2, 3’-cases of recombination are more frequent for *F*. *pratensis* chromosomes.

**Table S3c.** Distribution of values in plants of F_2_-F_4_ generations with respect to the number of recombined *L*. *perenne* and *F*. *pratensis* chromosomal arms with marker (M+)

| Type of chromosome recombination | Number of recombined arms | | |
| --- | --- | --- | --- |
|  | 0 | 1 | 2 |
| Arms recombined m+ *L*. *perenne* | 13 | 13 | 4 |
| Arms recombined m+ *F*. *pratensis* | 23 | 6 | 1 |

Pearson’s chi-squared 7.16. Probability 0.035. Significant difference between distributions; ‘0’-case of recombination is more frequent for *F*. *pratensis* chromosomes, while ‘1, 2’-cases of recombination are more frequent for *L*. *perenne* chromosomes.

**Table S3d.** Distribution of values in plants of F_2_-F_4_ generations with respect to the number of recombined *L*. *perenne* and *F*. *pratensis* chromosomal arms with marker (M+)

| Type of chromosome recombination | Number of recombined arms | | | | |
| --- | --- | --- | --- | --- | --- |
|  | 0 | 1 | 2 | 3 | 4 |
| Arms recombined m- *L*. *perenne* | 26 | 3 | 1 | 0 | 0 |
| Arms recombined m- *F*. *pratensis* | 17 | 8 | 3 | 1 | 1 |

Pearson’s chi-squared 7.16. Probability 0.078. No difference between distributions for *L*. *perenne* and *F*. *pratensis* chromosomes.
