# Supplementary figures and images for: Genome-dependent chromosome dynamics in three successive generations of the allotetraploid Festuca pratensis × Lolium perenne hybrid
Source: Protoplasma. 2014 Dec 6;252(4):985–96. doi: 10.1007/s00709-014-0734-9 (PMC4491343; doi:10.1007/s00709-014-0734-9)

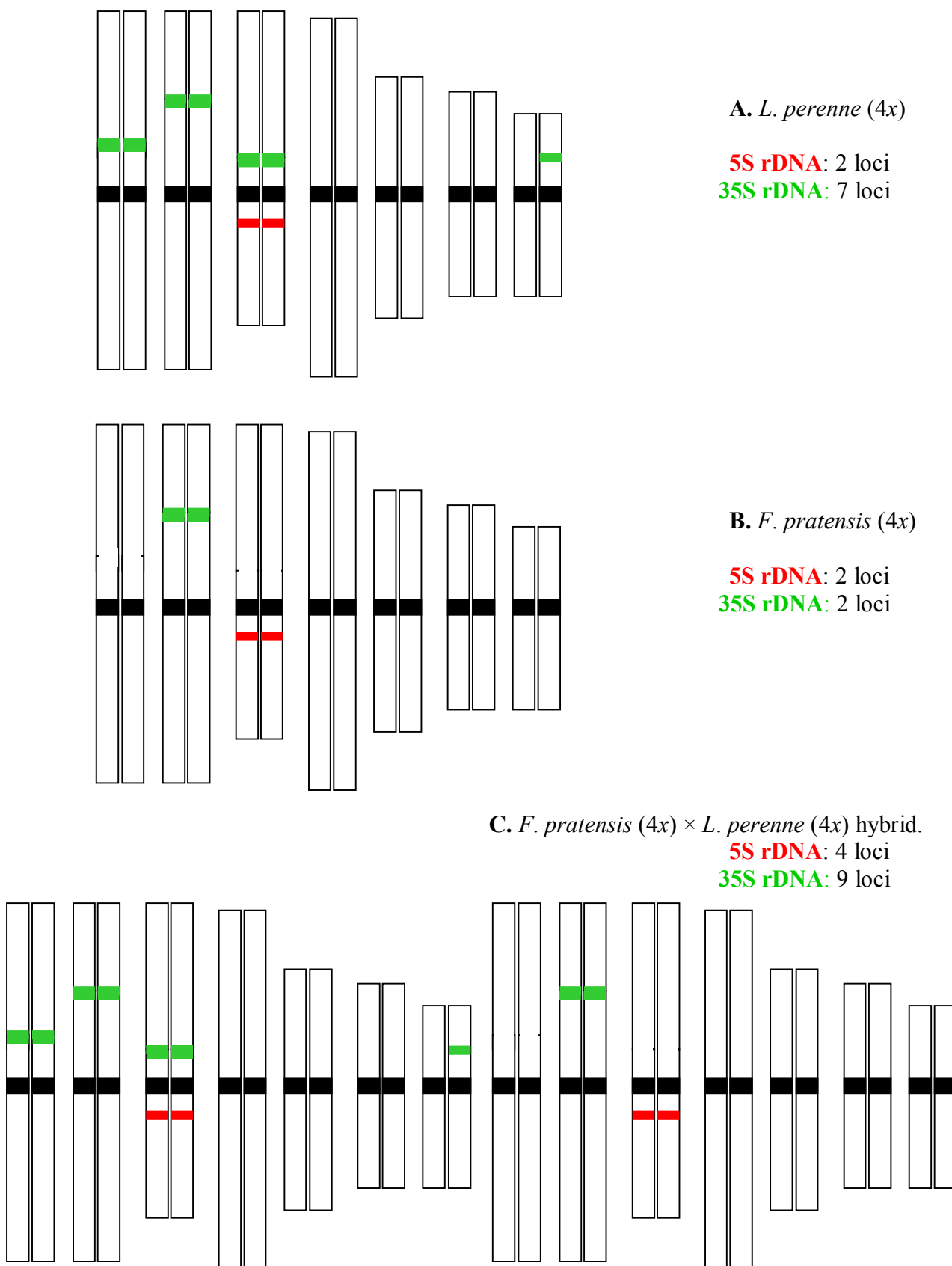

**Fig. S1.**

Supplement: Supplementary file 2 — Ideograms of the rDNA loci number and position in L. perenne (A), F. pratensis (B) and a hypothetical model of F1 karyotypes illustrating rDNA-bearing chromosome complement in F. pratensis (4x) × L. perenne (4x) hybrid (C). The rDNA loci pattern of L. perenne and F. pratensis is taken from Książczyk et al. (2010). (PDF 103 kb) [file 709_2014_734_MOESM2_ESM.pdf]

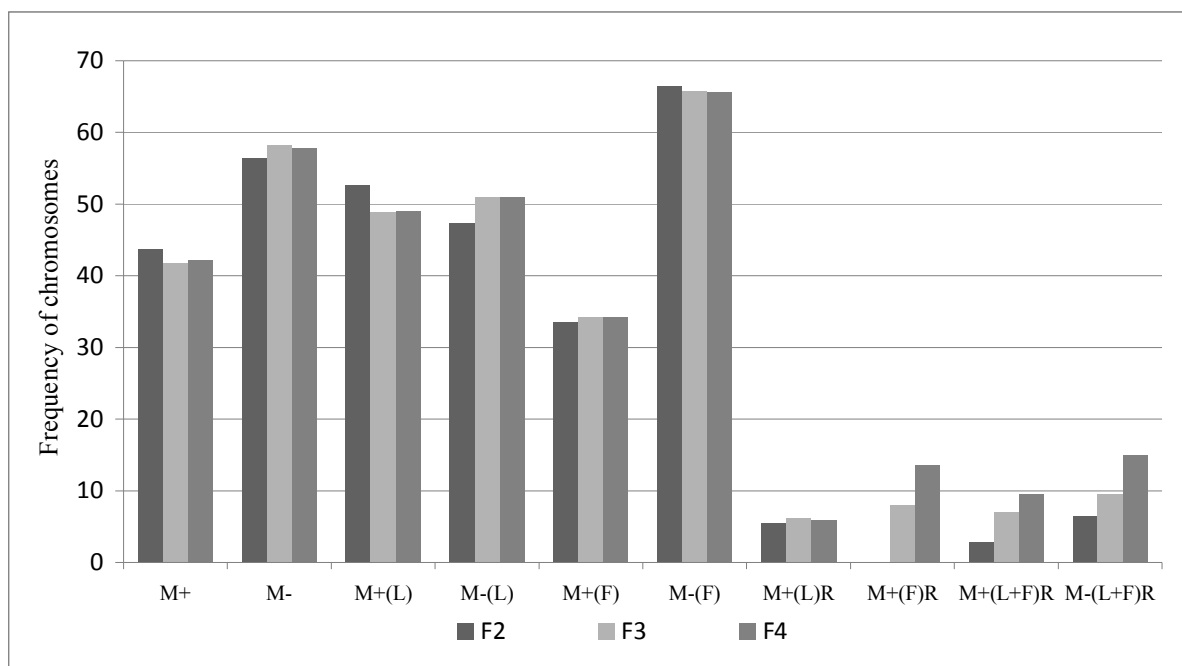

Fig. S2.

Supplement: Supplementary file 3 — Frequency profile of complete and recombinant L. perenne and F. pratensis rDNA-bearing and non-rDNA-bearing chromosomes in plants of the F2-F4 generations derived from F. pratensis (4x) × L. perenne (4x) hybrid. M+ Lp and Fp rDNA-bearing chromosomes, M- Lp and Fp non-rDNA-bearing chromosomes, M+(L) Lp and M+(F) Fp rDNA-bearing chromosomes, M-(L) Lp and M-(F) Fp non-rDNA-bearing chromosomes, M+(L)R recombinant Lp rDNA-bearing chromosomes, M+(F)R recombinant Fp rDNA-bearing chromosomes, M+(L + F)R total number of recombinant Lp and Fp rDNA-bearing chromosomes, M-(L + F)R total number of recombinant Lp and Fp non-rDNA-bearing chromosomes. (PDF 51 kb) [file 709_2014_734_MOESM3_ESM.pdf]
